# The future of medical and nursing education

**Published:** 2013-03-31

**Authors:** Peter Brooke

**Affiliations:** Norwich Medical School, United Kingdom

It would be very easy for this essay to be yet another paper on the problems facing doctors due to the European Working Times Directive, problems with nurse recruitment or the financial difficulties facing all new students. However, I wish to reflect on possible future improvement and curricular changes within both medical and nursing education.

The last twenty years have seen the development and rise in popularity of Problem Based Learning (PBL) in the UK, Canada and medical schools elsewhere across the globe. So too have nursing courses across the UK embraced Enquiry Based Learning (EBL), a similar self-directed learning technique. Both PBL and EBL are considerably cheaper to operate compared to traditional courses. Nurses and doctors have to learn many similar topics over a broad range of areas, albeit to a different depth. It is suggested here that future course curriculum development may see moves to hold some teaching jointly with both nursing and medical students. The differences between nurses and doctors are vast, and it is not suggested here that nurses should attend all medical lectures, or vice versa, simply that there may be a benefit to holding some teaching jointly.

The most obvious argument to make against this suggestion is that of knowledge depth. Arguably doctors are required to learn Medicine in far greater depth than nurses. Equally, there are areas of nursing that doctors remain ignorant of. What need is there for doctors to sit in on manual handling lectures, or for nurses to study the pharmacokinetics of Warfarin? Even if common ground can be found, or lectures rewritten to give a joint introduction to both professions, is there any real benefit to doing so? By teaching both groups together for some areas, an element of camaraderie and shared learning may occur with both sides enriching the other to become something more than the sum of their parts.

The simplest argument to make in favour of some joint teaching is that of cost. As modern universities come under increasing financial pressure, savings must be found wherever possible. Lectures or tutorials on general topic introductions or practical skills such as inhaler technique or blood glucose monitoring could be held jointly. Efficiency savings could encourage universities to consider joint teaching and even a relatively small number of joint sessions would result in substantial savings.

Inter-professional Learning (IPL) is a globally occurring phenomenon aimed at encouraging healthcare students to work cooperatively together. It is an attempt to foster inter-professional working relationships by increasing the level of understanding around professionals’ roles within a team. Sadly, IPL is generally not well received by students, commenting that it feels “forced” or “artificial”. In the author’s experience, students from the professions involved do participate, but occasionally appear to have professional stereotypes reinforced rather than rebuffed. Do nurses and doctors even want to study together or could such an approach widen the gap between “us” and “them” to new levels of mistrust?

By conducting general teaching together, students would have opportunities to socialise and learn together in a normal environment. Artificial cooperation tasks would be unnecessary as students discovered their professional identities over the course of their degree, while studying together would encourage teamwork and understanding between courses. An added benefit of collaborative working is that it may start to break down the “us” vs. “them” attitudes that are common within both professions. By learning together and gaining awareness of what both professions can do, tolerance and acceptance may start to replace mistrust and judgement.

To develop this idea further, PBL and EBL groups could merge and group learning sessions would facilitate closer cooperation between groups, nurturing professional relationships and friendships. Logistically this may be harder as both groups of students would also need time to discuss the more course-specific learning. However, it is suggested that if this were possible, the inter-professional educational and professional benefits of such a technique would have a large impact on the medical community.

Although it is possible to argue that this would hinder inter-professional working, the same can be said of the current IPL system. This suggestion would clearly have benefits in terms of cost savings to universities, natural development of inter-professional relationships and encourage teamwork and teaching within student groups. Although clearly both courses do have different teaching goals and require different levels of teaching in many areas, there is no obvious reason why some teaching should not occur jointly, given the existence of common strands that tie both courses together.

